# Asylum in Politics—Medical Legislation—Medical Organization, Etc.

**Published:** 1895-04

**Authors:** Daniel Parker

**Affiliations:** Calvert, Texas


### Correspondence.

### Asylums in Politics—Medical Legislation—Medical Organization, etc.

LETTER FROM DR. DANIEL PARKER, CALVERT, TEXAS.

*Editor Texas Medical Journal and Readers thereof:*

I am but little given to obtruding my opinions on the brethren, not for the reason that I do not think well of them, but for the reason that the presumption is that they are of not much consequence to anybody else; but just now several topics, semi-professional, are warring in my mind for utterance, and I feel somewhat as I imagine the zealous convert does when called to preach.

In the first place, I want to say, that I do not like the way the officers of our insane asylums are changed quadrenially; no, not a bit. It is all wrong. No doubt Gov. Culberson will feel badly about it when he finds out that I think so, but I can't help it. That is the way it looks to a plain, single-barreled doctor, and I had as well say so. Personally I do not know whether Drs. White and Preston were properly equipped to fill the positions they have just vacated or not. I believe they were, as they made a good showing and all men speak well of them. If they were not, it certainly was a great wrong that they should be retained so many years to act for those unfortunates who are incapacitated to act for themselves. If, however, as I believe, they were well qualified to discharge the duties of the position they held, what good reason can be given for removing them and filling their places with men without special training for the work? None in reason or common sense. A man who would conduct his private business in such a manner, that is, discharge competent and experienced men and place inexperienced men in charge, would not be able to get credit among prudent business men for goods enough to start a peanut stand. It was wrong in "box car letters," if the governor did do it. I do not question the character or ability of the gentlemen appointed. I have met only one of them, and he impressed me as being not only a man of ability, but a man of affairs; good timber *to make* a superintendent out of; but he is not the man I take him to be, if he would claim to be as well qualified to fill the position he now holds as the retiring superintendent was. Anyway, if he does as well as I think he will, I hope, when the present administration changes, the coming man will not find it expedient to trade in the afflictions of the demented and oust him, to make place for a tyro. No doubt, there are a hundred physicians in Texas who are good material for superintendents, but at present we have places for only three. Let us have three good ones, and when they become skilled in the work let us retain them "during life and good behavior." This may not be good politics, but it is good policy, good sense, and humanity, and nothing else is. The present system of appointing is wrong. The governor is no better qualified to appoint superintendents of asylums than Dr. McLaughlin is to appoint judges of the courts, and it is no more fitting that he should do it. Neither the welfare of the sick, or the appointment, or official tenure of medical officers should be subject to the needs or caprices of executive aspirants. The profession are so accustomed to being ignored in such matters that we take it as a matter of course and let everything go by default. I believe we have a duty to perform. Let us all speak out.

Now a word on the well worn subject of State Medical Association. The whole trouble lies in the immense size of our State. It requires no prophetic power to say that comparatively few physicians north of Corsicana will attend an Association at Galveston, Austin or San Antonio, and that but few south of Waco will attend at Dallas or any north Texas town. There are many willing, but they just cannot afford it, and that settles it. If this statement is true, what sort of an attendence would there be if it was permanently located at Galveston or Austin, as advocated by some. It would immediately become a State Association in name only, and if it succeed at all, would become simply a local organization. No, brethren, keep the thing on wheels. Give us a sniff of salt water one year, the aroma of the pine and jessamine another, then a whirl over the breezy and boundless prairie, and finally lest the fires of our State pride should burn low, let us visit the matrix of Texas liberty, the Alamo, and worship at the shrine of valor as well as of Æsculapius. Texas is too big to take in all at once, but we can "hug her in spots," as the man did his big wife, and that is the only way to have a *State* Association. Like all the rest, I have a plan that is a great improvement on our present organization, but have no time to develop it here and now.

Right here I want to sandwich in a little family matter where nobody but kin folks will see it. I have had an illusion dispelled, I might say an idol shattered. On the first of the year I received a document signed by the great surgeons of a great railroad corporation appointing me local surgeon of said corporation, which, after enumerating the duties I was expected to perform (which were principally to hustle unfortunates off to the general infimary as quick as possible), concluded with the statement, that in consideration of these services, I would be granted the princely compensation of an annual pass over the road, and apparently to make it as worthless as possible, it was specially stipulated that said corporation was not responsible for the life, limb, or property of any one using it. The pass was enclosed. Well, I was considerably taken back. I did not know whether the great surgeons had reason to believe that I was just fit for an emergency dresser, and had nothing to do but ride up and down the road for pay, or whether there was really some magic potency in a pass, as I had never seen one before; so I put it aside, turned it over occasionally, and used all the theurgic phrases I knew, such as "hocus pocus," "presto, change," etc., until I was convinced it was no kin to Aladdin's lamp, but was just an ordinary piece of paste board, and that if I got anything out of it, I would have to ride on the "steam cars" to get it. This conclusion having been reached, I informed the generous gentleman that however true it might be, I was not willing to admit that my service were worth nothing, that I would accept the position with thanks upon the basis of a reasonable fee for actual services rendered; otherwise I would return the pass, etc. The result was, we didn't trade, and the pass was returned.

Now for the illusion. I had thought that it was a compliment —not a very large one, it is true, but still a compliment—to hold the position of local railroad surgeon, and to use a mercenary phrase, there was some money in it, just a little, but enough to make it respectable. Also, when I heard or read of railroad surgeons traveling singly or in bodies over the country, I said to myself, it is well, these great men need rest, and as is fitting, the lucrative character of their engagements with generous corporations enables them to indulge in much needed relaxations, when lo and behold, the poor devils were riding out their doctor bills, out collecting, you might term it.

Now, seriously, while this may seem to be a personal matter and consequently not a proper subject for publication, I take it for granted that the proposition submitted to me is the usual one, and I respectfully submit that it is so little short of a professional insult, that any man who has proper respect for the profession ought never to make or accept it.

It could hardly be expected that such a rambling letter as this could be brought to a close without some reference to the trite subject of medical legislation. In common with all the scribes I believe in medical legislation, but I have never seen any proposed yet that suited me. I believe that every one who has arrived at the age of discretion should be allowed to have any man, or woman, that he chooses to give him his medicine, and that he should be allowed to take any kind of medicine or nostrum he wants to. That is democracy, and I believe in democracy in medicine, religion and politics. The law should only protect the public and citizen against imposition. No man should be allowed to practice medicine or advertise to cure without giving his name, degree, if he has any, system of practice, college at which he graduated, and date of graduation. This should all be conspicuously displayed. If he is not a graduate, let him display his name, system of practice, and afterwards write Dr. S. C. (doctor so-called). Then a man knows the brand, and takes his choice, whether he pays his money or not. The same principle should apply to the sale of medicines. Those who so desire should be provided with an opportunity to purchase and fill their stomachs with all the vile nostrums they can hear of. The law should simply provide them with reliable information as to what they are taking. If a man wants ''Bile Beans,'' ''Peruna,'' or ''Sheep Saffron,'' he ought to have it. Just let him know that bile beans are composed of such and such proportions of podopholin, gambogue, etc. Peruna, of stump water, R. G. whisky and sugar, and sheep saffron, of so many pellets to the pint, and then let him revel in well informed but unobstructed taste. Such a law could be passed and enforced. It would be reasonable, democratic, and goes far enough for a Texan. It would do more towards the suppression of quackery and the sale of patent medicines than all the stringent prohibitory laws that could be placed on the statute books.

The people resent having their private affairs meddled with. Just give them light and they can be trusted to take care of themselves, and light is just what quackery tries to avoid.

Sincerely yours,

DANIEL PARKER.

Calvert, Texas, March 16, 1895.

P. S.—Since writing the foregoing I have read Dr. Orr's article in the JOURNAL for March. I am glad to see it. Let us have firing all along the line. I did not know before that any law except that of economy, good business and common sense, had been violated in the asylum appointments, and I have no doubt ninety per cent. of the people are as much in the dark as I was. Let us have "more light." D. P.

